# Distributive justice and value trade-offs in antibiotic use in aged care settings

**DOI:** 10.1007/s40592-024-00191-5

**Published:** 2024-07-11

**Authors:** Jane Williams, Sittichoke Chawraingern, Chris Degeling

**Affiliations:** 1 Australian Centre for Health Engagement, Evidence and Values (ACHEEV), School of Health and Society, University of Wollongong, Wollongong, Australia; 2 Sydney Health Ethics, School of Public Health, The University of Sydney, Sydney, Australia

**Keywords:** Antimicrobial resistance, Distributive justice, Value trade offs, Antimicrobial stewardship

## Abstract

Residential aged care facilities (RACF) are sites of high antibiotic use in Australia. Misuse of antimicrobial drugs in RACF contributes to antimicrobial resistance (AMR) burdens that accrue to individuals and the wider public, now and in the future. Antimicrobial stewardship (AMS) practices in RACF, e.g. requiring conformation of infection, are designed to minimise inappropriate use of antibiotics. We conducted dialogue groups with 46 participants with a parent receiving aged care to better understand families’ perspectives on antibiotics and care in RACF. Participants grappled with value trade offs in thinking about their own parents’ care, juggling imagined population and future harms with known short term comfort of individuals and prioritising the latter. Distributive justice in AMR relies on collective moral responsibility and action for the benefit of future generations and unknown others. In RACF, AMS requires value trade-offs and compromise on antimicrobial use in an environment that is heavily reliant on antimicrobial drugs to perform caring functions. In the context of aged care, AMS is a technical solution to a deeply relational and socio-structural problem and there is a risk that carers (workers, families) are morally burdened by system failures that are not addressed in AMS solutions.

**Supplementary Information:**

The online version contains supplementary material available at 10.1007/s40592-024-00191-5.

## Introduction

Antimicrobial resistance (AMR) occurs when pathogens such as bacteria become less susceptible to antimicrobial drugs such as antibiotics. AMR is recognised to be an increasingly urgent public health problem—a ‘super-wicked problem’—that requires committed multisectoral action to mitigate its harms (Littmann et al. 2020; World Health Organisation 2021). A major cause of AMR is the misuse and overuse of antimicrobial medicines in human healthcare. As antimicrobial drugs become less effective, infections that were previously easy to prevent or treat can cause greater morbidity and mortality, increasing the burden of disease and cost of healthcare. Examples of common infections that are currently difficult to treat with antibiotics include foodborne campylobacter and salmonella infections, and hospital acquired infections such as Clostridium difficile and vancomycin resistant enterococci, and there is a growing list of threat (CDC 2022). 4.95 million deaths were associated with AMR in 2019 and this is forecast to rise to as many as 10 million deaths per year by 2050 (Murray et al. 2022; Varadan et al. 2023).

As an emerging issue in clinical practice and public health, AMR is simultaneously a very straightforward concept—harmful bacteria exposed to chemotherapeutic agents often become resistant to them over time—and a slippery set of context contingent problems. AMR can emerge in an individual or in the population. For example, an infection such as a urinary tract infection (UTI) repeatedly treated with the same drug can become resistant to that drug over time. In this situation, harms accrue to the individual with the recurring infection as it becomes more difficult to treat. At the population level, drugs that used to cure common infections such as sepsis and sexually transmitted infections are less effective with ongoing use. This is because of the increased prevalence of genes that confer resistance in current strains of pathogens, and these mobile genetic elements can spread across, within and between both microbial and host populations (Moser et al. 2018; Acman et al. 2022). Tensions between individual and population effects are a mainstay of public health ethics, particularly when a public health outcome is the result of many individual clinical interactions. Thinking about AMR also involves difficult contextual and temporal considerations around the goals of antimicrobial therapy and current and future harms and benefits.

Antimicrobial stewardship (AMS) is one of many multisectoral approaches to reducing AMR harms. It is a largely technical solution that aims to minimise the inappropriate prescription of medicines such as antibiotics and might include measures such as prescriber and patient education, restricting the availability of key antimicrobials, and diagnostic requirements (Fishman 2006). One site of AMS practice in Australia is residential aged care facilities (RACF), a setting associated with high antibiotic use. 8–9% of RACF residents are receiving antibiotics at any given time (Aged Care Quality and Safety Commission, no date). Approximately 50% of these prescriptions are judged to be inappropriate; with antimicrobial misuse in the treatment of urinary tract infections and respiratory tract infections a common occurrence (Sluggett et al. 2021). People who live in RACF have greater vulnerability to infections than the general population, a higher burden of complex diseases, and, as they near the end of life, higher care needs (Aged Care Quality and Safety Commission, no date). Rather than allow indiscriminate or empirical antibiotic use in RACF, AMS in these settings involves a number of practices designed to prevent and more effectively treat infection, such as enhanced infection control protocols and requirements for pathology testing and confirmation of an infection before issuing antibiotics. Studies have shown that prescribers in an RACF setting experience social pressure from RACF staff and families to prescribe antibiotics outside of AMS guidelines (Lim et al. 2014; Hall et al. 2022).

Underpinning AMR problems and AMS responses is scarcity. The drugs we have are increasingly ineffective and the outlook for timely new alternatives is grim. Who should and should not access antimicrobials under what circumstances is a distributive justice issue and various suggestions and solutions have been offered. In countries with well-resourced healthcare systems, justice involves judicious use of antimicrobials now so that they are available for future generations. On the global scale, however, distributive justice means regulating antimicrobial drugs and making them available to people who cannot access them appropriately (Littmann and Viens 2015). Others argue that increasing access to people in low-income countries has the potential to worsen effectiveness, on the assumption of higher consumption across the board. Higher illness thresholds for access can counter this threat at the cost of increased individual suffering (Millar 2011). Distributive justice arguments in the literature are big picture arguments, but an emerging literature indicates that AMR and AMS are highly context dependent (Hinchliffe et al. 2021; Degeling and Hall 2023; Johnson and Matlock 2023). Here we examine distributive justice in the aged care setting.

## A dialogue group study

We conducted a qualitative research study that used dialogue groups to explore views on antibiotic use and AMS of family members with an older parent receiving aged care. We ran eight 90-min online dialogue groups, each containing six participants. Dialogue groups are a qualitative research method where a group is given a fictional, realistic, scenario to discuss. Over the course of the discussion key characteristics of the scenario are changed to reset key normative considerations; the aim of the method is to draw out values that underpin responses to a particular scenario (Banks et al. 2006). The scenario used in this study was of a 75-year-old woman with mild dementia who lives in a residential aged care and has recurring infections; UTIs where AMR harms accrue to the individual over time, and a drug-resistant E. coli infection that, with poor hygiene practices, could result in population-level AMR harms (see Online Appendix 1). We have reported on that study and its methods elsewhere (Degeling et al. 2023). The purpose of this paper is to examine the value trade-offs that participants raised as they worked through their responses to the scenario put to them, an analysis that was out of scope of earlier work. Value trade-offs are navigations that occur when several things are valued at once. They do not reflect value disagreements, rather the construction of a way of thinking about an issue in the face of multiple, important but incommensurate things. We use participants’ experiences of working through a tricky and familiar situation to further investigate the type of problem that is AMR/AMS in the context of older populations and RACF settings. The literature suggests that publics have an awareness of AMR but do not understand it well (McCullough et al. 2016). Our study did not seek to test understanding of AMR or AMS, rather its primary aim was to understand under what circumstances (if any) family members advocated for antibiotic use outside of AMS guidelines. We did not tell participants that they should think about antibiotic use in the context of individual and/or collective responsibilities to mitigate AMR, but they might reasonably have inferred this from the scenario preamble.

Underpinning the value trade-offs we describe were some patterns in how participants treated the problem of potentially inappropriate use of antibiotics in RACF. First, they were much more likely to focus on standards of care and diminishing effectiveness of antibiotics in the individual than the potential impacts of increasing AMR for the population. Second, participants expressed beliefs about antibiotics as a treatment method (e.g. ‘*I believe that antibiotics should be used to save life and limb’* or *‘young people should avoid antibiotics’*). Again, this belief was focused more on the perceptions of antibiotics causing unspecified harms to individuals rather than a reference to broad societal threat. The dominant consideration of antibiotic use as an individual issue shaped how participants grappled with value trade-offs related to their distribution as they discussed the scenario put to them in the study.

The trade-offs we describe here parallel classic health economics cost benefit analysis problems, where benefits, harms, temporal shifts and quality of life are measured in order to explain decisions about health behaviour. Our study did not attempt any measurements, nor did we ask participants to engage with trade-offs; rather the trade-offs arose organically in discussion about judicious antibiotic use. We describe and analyse trade-offs in the specific context of AMR/AMS in residential aged care facilities and conclude with remarks about the generalisability of the construction of distributive justice in AMR/AMS problems.

## Value trade off 1: ‘but it’s my mum’, or antibiotics as care

Participants agreed that avoiding antibiotic use was good in theory, generally for somewhat nebulous reasons. Almost all participants supported withholding antibiotics until a diagnosis of infection was confirmed by laboratory testing. However as the scenario they were considering became more complex and the character at its centre gradually more vulnerable to discomfort, that position shifted to one of prioritising antibiotic centred care even if that meant arguing for drug use that fell outside of AMS guidelines. Participants felt they had an advocacy role with their parents and that withholding medication could potentially lead to a longer period of discomfort, which they agreed should be avoided in an older person. This trade-off was explicit—e.g. “*I know it’s not right but it's my mum”*—pitting the care of a loved one against a perceived public good.

Favouring an identifiable individual over a statistical other (or the general public) in the context of scarcity is a well-established idea though its moral legitimacy is debated (Daniels 2012). In instances of immediate scarcity it can influence the favouring of one person’s claim over another’s because of a special relationship when all else is equal; some consequentialists aside, few would argue that it is not morally permissible to favour the life of a loved one over the life of a stranger. AMR is not usually a problem of immediate scarcity in the context of RACF in Australia, and the relationship between an adult child and their vulnerable elderly parent is not simply one of an identifiable individual but rather more complex and meaning-laden. AMS in the context of aged care points to a particular kind of distributional justice. In order to preserve antimicrobial drugs for future use, and because of the inherent uncertainties that can surround the diagnosis of common infections in elderly populations, people in aged care have higher barriers to their access. The extent to which this was an acceptable trade-off depended primarily on the extent of the discomfort the patient was experiencing. A small amount of discomfort while waiting for diagnostic confirmation of infection was acceptable, but each participant had their own threshold for when ‘withholding’ antibiotics in conditions of uncertainty became unacceptable.

Advocating for antibiotic use that fell outside of AMS practices was construed as an expression of love and care towards a vulnerable parent who had loved and cared for the now-adult child in the past. This meant that antibiotics held a social and moral importance beyond simply being a therapeutic drug. The value trade-off for most was a fairly simple calculation—a loved person deserved care irrespective of the population harms to which that care could contribute.

Antibiotics are commonly prescribed for palliation at the end of life, further complicating ideal AMS norms of the provision of antimicrobials as curative and necessary. As the fictitious character in the scenario became more ill and her infection intractable, value trade-offs shifted from weighing up the importance of the individual vs. population goods to weighing up the value of a longer life vs. a better life. Here, the decision was not so clear cut and there was a tension between expressions of care associated with keeping a loved one alive for as long as possible, minimising suffering, and what constituted worthwhile life extension. Balancing value trade-offs about end-of-life care are an increasingly common experience in high-technology palliative settings but not central to ethics debates about AMR and AMS. In an examination of vulnerability and its relationship with AMR, Alex Broom and colleagues argue that in addition to vulnerability being an outcome of AMR it also contributes to the production of AMR (Broom et al. 2023). We suggest that it is vulnerability and an obligation to care that drives the value trade-offs that people navigated, rather than thinking about antibiotics as a value-neutral technological fix.

## Value trade-off 2: ‘we’ll just have to make new ones’, or intergenerational justice

AMR problems are often presented as future problems both in terms of their impacts and potential solutions. As part of participants’ navigation of value trade-offs, they expressed hope that new drugs would be developed that would make pathogens easier to manage in the future. This meant that they were able to discount the worrisome future scenarios they had heard about in favour of an imagined, more positive, one and couch their preference for antibiotics use now in light of that imagined future.

Discounting the importance of an uncertain future in favour of a knowable present has long been the domain of economists (Kay and King 2021) and debated by ethicists (Rawls 1971; Parfit 1984); their role in the value trade-offs of people faced with AMR dilemmas is worth highlighting (Rid et al. 2019). Most of the dialogue group discussion treated temporal separation from the current scenario in the same way as they did the problem of special relationships. That is to say, a vulnerable known older person who could receive some comfort from antibiotics now was valued more highly than a scarcely imagined future population in need. Only one participant (of 48) made a connection between current and future needs of *known* people when he observed that giving antibiotics to his mother now might mean that there were none available for him or his child when they were older. This comment did not gain traction in the group discussion, suggesting that intergenerational justice arguments that are common in the literature were not instinctively appealing to participants. Discounted futures meant that the current problem had primacy in value trade-offs. Had the group been forced to choose between treating their parent and treating their child now, we suspect that the conversation would have been very different. Discounting even a difficult and clearly imagined future scenario was easier.

Acting now to mitigate future harms is an idea that is front of mind for many in the context of other threats, and we can compare AMR threats to those of climate change. We see and experience the harms of climate change now and know that climate change harms will become worse over time. We know that our current actions will contribute to a future that is better or worse both for people with whom we have a special relationship and statistical others. We know that we should sacrifice some of our current comforts to make future lives less uncomfortable. And, as with AMR, we can assume that future lives will not be equally affected and that people with more precarity will be worse off. With both AMR and climate change, however, it is tempting to make exceptions to planned and well-intended sacrifices in order to prioritise current relationships and comfort.

The focus of AMS as presented in the scenario (managing access to therapeutics now to preserve efficacy) was insufficient for participants in this study, and they sought to reframe the problem. AMR would be less of an issue, and the trade-offs faced less fraught, they argued, if there was less infection in people living in RACF. Using different empirical evidence and theoretical resources, others (Millar 2011; Chandler 2019; Willis and Chandler 2019; Broom et al. 2021) argue the same point: that it is the determinants of infectious disease that need our attention at least as much as how to manage scarce cures. There are both physiological and social reasons for increased prevalence of infection in older people (Aged Care Quality and Safety Commission, no date) and participants argued that it was likely that poor care in the RACF model contributed to infection. A focus on how (poorly paid and precariously employed) aged care workers could be doing more to avoid infection in residents meant that participants were able to imagine another alternative future. Along with a future where new effective antimicrobials were available was an imagined future where better standards of care would mean that infection was much less common. The tricky problems of distributive justice could thus be avoided.

## Discussion

In her reconfiguration of commons problems and solutions, Elinor Ostrom highlights the importance of context and argues that:

*‘Instead of there being a single solution to a single problem […] many solutions exist to cope with many different problems. Instead of presuming that optimal institutional solutions can be designed easily and imposed at low cost by external authorities […] ‘getting the institutions right’ is a difficult, time-consuming, conflict-invoking practice […] that requires reliable information about time and place variables as well as a broad repertoire of culturally acceptable rules’.* (Ostrom 1990), p. 14

AMR and AMS responses are understandably presented as systems problems that are global in scope, but their effects also accrue to individuals. Our dialogue groups with adult children of parents receiving aged care indicated that distributive justice, for them, came down to the importance of a loved individual receiving care in the present. There are possible cultural and structural explanations for this. It could be that we are, in Australia, so accustomed to health systems working that we struggle to imagine a future where they do not. The current availability of antibiotics and the hidden work they do in the background of our daily lives is such that the ramifications of non-availability seem relevant only to an ill person needing individual care in the present. For many people brought up in a world invested in hyper sanitation, the idea of leaving an infection untreated, or using antibiotics to suppress rather than cure infection, is difficult to stomach. In addition, the introduction of AMS programs in health care are deeply embedded in and dependent on local political priorities and social and economic structures (Broom et al. 2021). Modern economies and many of their composite systems rely on antibiotics to perform as a form of infrastructure that acts as a proxy for higher standards of care, hygiene, and infection control (Chandler 2019). Arguably, aged care has organised around staffing levels and models of care that depend on ready access to antibiotics, rather than more financially costly and scarce human resources (Hall et al. 2022; Australian Government Department of Health and Aged Care 2023). These contextual features of antibiotic use in RACF are significant to AMS because they have exacerbated a dependence on antibiotics as a social care norm.

The primary aim of AMS is to assist with prescribing decisions so that therapy is more effective and safer for people who need it. Preserving the efficacy of antibiotics for as long as possible for everyone (now and in the future) is a secondary effect. In aged care, AMS guidelines provide clear rules about when the provision of antibiotics is warranted, removing or alleviating decision-making dilemmas faced by aged care and healthcare workers. However the guidelines exist in an environment that has long been dependent on antibiotics use, and where the population we studied perceived advocating for their use as an expression of care. AMS guidelines, institutionally developed and carefully considered and applied, could be an example of what Broom et al. call for in a solidarity-based approach to AMR (Broom et al. 2021). Despite a strong institutional attempt to provide leadership, however, adult children of parents receiving aged care felt deeply conflicted as they navigated trade-offs when considering their parents’ needs and advocating for prescribing outside of guidelines. The provision of guidelines did not address the structural and moral burdens that participants described.

Central to broader discussions of the distributive ethics of AMR is the idea that we are all morally responsible for supporting practices that contribute to corporate public goods. Corporate goods are those goods that benefit a community or society rather than or in addition to a collection of individuals (aggregative goods) (Widdows and Cordell 2011). In the case of aged care, this can mean sitting uncomfortably with more or higher barriers to access, or it can mean the practical expression of moral commitments to future generations by declining, for e.g., palliative antibiotics. These options may be more acceptable in the abstract than in practice, which has implications for how ethicists think about just distribution in the context of aged care.

The infrastructural role played by antibiotics and a rejection of the commitments that underpin AMS leads to increased moral burdens on carers and pressure on people working in aged care to ensure out-of-guideline access to antimicrobial drugs (Hall et al. 2022). As we have shown, in the context of aged care, AMS is a technical solution to a deeply relational and socio-structural problem. An additional shortcoming of technical AMS solutions to AMR problems is that it has the capacity to paper over or compensate for gaps in care, and other modifiable determinants of infection. This means that instead of the aged care sector being forced to address costly and difficult issues such as quality of care and staffing models, antimicrobials are employed to back up faltering systems. As ethicists consider distributive justice solutions to AMR problems, there is a risk that individuals with parents receiving aged care are morally burdened by system failures that are not addressed in AMS solutions. We acknowledge that increasingly granular attention to context can be unhelpful when the goal is a long-term and wide-ranging good. However we consider that AMS attempts that are premised on structural shortcomings—e.g. antibiotic use as a proxy for more resource intensive care—are insufficient. Plans for just distribution of antimicrobial therapies ought not to happen in isolation but must attend also to the also wicked problem of the determinants of infection.

Our context specific examination of trade-offs in the distribution of antimicrobial drugs supports Ostrom’s claim: that attempts to manage scarce common goods need to be keenly attentive to context and culture if their design and implementation are to be successful.

## Supplementary Information

Below is the link to the electronic supplementary material.

Supplementary file1 (DOCX 16 KB)

## Data Availability

Data are not available due to privacy constraints, in particular the risk of participant identification.
