# Supplementary material for: Distributive justice and value trade-offs in antibiotic use in aged care settings
Source: Monash Bioeth Rev. 2024 Jul 11;42(Suppl 1):41–50. doi: 10.1007/s40592-024-00191-5 (PMC11850501; doi:10.1007/s40592-024-00191-5)
Supplement: Supplementary file 1 — Supplementary file1 (DOCX 16 KB) [file 40592_2024_191_MOESM1_ESM.docx]

## Appendix 1: Dialogue group scenario

### Backstory

Mary is a 75-year-old female resident in a residential aged care facility (RACF). During a visit Mary’s daughter becomes concerned about how lethargic Mary is. She hasn’t been able to visit her mother for the last few days and she tells the nurse on duty about her concerns.

The nurse does not know Mary very well. She looks at Mary’s care notes and can see no-one has raised prior concerns. The nurse takes Mary’s temperature and finds a low-grade fever. Mary is perhaps more withdrawn than expected, considering that she has visitors.

Mary has a history of urinary tract infections (UTI) with similar symptoms which soon became much worse, but got better after a course of antibiotics. Because of this history Mary’s daughter asks the nurse whether antibiotics should just be given immediately because last time Mary had a UTI she became very ill.

The nurse tells Mary’s daughter that urine should be collected and tested before antibiotics can be given to make sure they are necessary.  She tries to reassure her that the RACF care providers will keep an eye on Mary and call her once the urine sample has been tested.

### Questions posed to participants

The participants were progressively given the following scenarios and asked what they thought each actor (family members, RACF nurses, doctors) should do at a number of timepoints.

They were also asked what they think is reasonable for society to expect of older people in relation to antibiotic stewardship

### Scenario 1

The RACF nurses are not able to collect a clean urine sample in the following 24 hours. This delays diagnosis because a test cannot be done. The doctor has been contacted and in the absence of confirmatory pathology testing is continuing to not prescribe antibiotics and watch and wait because Mary is not developing worse symptoms.

### Scenario 2 (Continuation of scenario 1)

24 hours later a urine sample is collected but does not confirm the presence of a UTI. Antibiotics are withheld and Mary is to continue to be carefully monitored with efforts made to increase her drinking

### Scenario 3 version A (Continuation of scenario 2)

Mary recovers over the next few days under the care of RACF staff without antibiotics

### Scenario 3 version B (Continuation of scenario 2)

Instead of getting better Mary develops a high fever and begins to be in pain when she urinates. Another urine sample is sent for testing. Mary is prescribed antibiotics by the doctor while the results are pending. But develops severe side effects such as vomiting and diarrhoea before the results are known.

### Scenario 4 (Continuation of scenario 3 version B)

The tests diagnose a multi-resistant E.coli bacterial infection – limiting therapeutic choices to 3 antibiotics. The antibiotic prescribed is changed to one Mary tolerates and she recovers over the following week.

### Scenario 5 (Continuation of scenario 4)

3 months later Mary develops another UTI. Testing finds the same E.coli but it is now highly resistant so that it is only susceptible to two antibiotics: the one that made Mary extremely physically sick last time; and one that is hepatotoxic and nephrotoxic (requiring hospitalisation for administration). This second antibiotic only inhibits but does not kill the E.coli causing the infection. Mary is likely to continue to get infections every few months that will need hospital stays and it is likely that the E.coli will develop resistance to this last line treatment.
